# Oberlin partial ulnar nerve transfer for restoration in obstetric brachial plexus palsy of a newborn: case report

**DOI:** 10.1186/1749-7221-1-3

**Published:** 2006-09-29

**Authors:** Koji Shigematsu, Hiroshi Yajima, Yasunori Kobata, Kenji Kawamura, Naoki Maegawa, Yoshinori Takakura

**Affiliations:** 1 Department of Orthopaedic Surgery, Nara Medical University, Nara, Japan

## Abstract

An 8 month old male infant with Erb's birth palsy was treated with two peripheral nerve transfers. Except for rapid motor reinnervations, elbow flexion was obtained by an Oberlin's partial ulnar nerve transfer, while shoulder abduction was restored by an accessory-to-suprascapular nerve transfer. The initial contraction of the biceps muscle occurred two months after surgery. Forty months after surgery, elbow flexion reached M5 without functional loss of the ulnar nerve. This case demonstrates an excellent result of an Oberlin's nerve transfer for restoration of flexion of the elbow joint in Erb's birth palsy. However, at this time partial ulnar nerve transfer for Erb's birth palsy is an optional procedure; a larger number of cases will need to be studied for it to be widely accepted as a standard procedure for Erb's palsy at birth.

## Background

In 1994, Oberlin et al. [1] described a new technique of partial ulnar nerve transfer to the biceps muscle nerve for restoration of elbow flexion in traumatic C5-C6 avulsion of the brachial plexus in adult. We report treating an eight month old male infant without C5 to C6 function by an Oberlin's partial ulnar nerve transfer and an accessory-to-suprascapular nerve transfer.

## Case presentation

**Figure 1:**
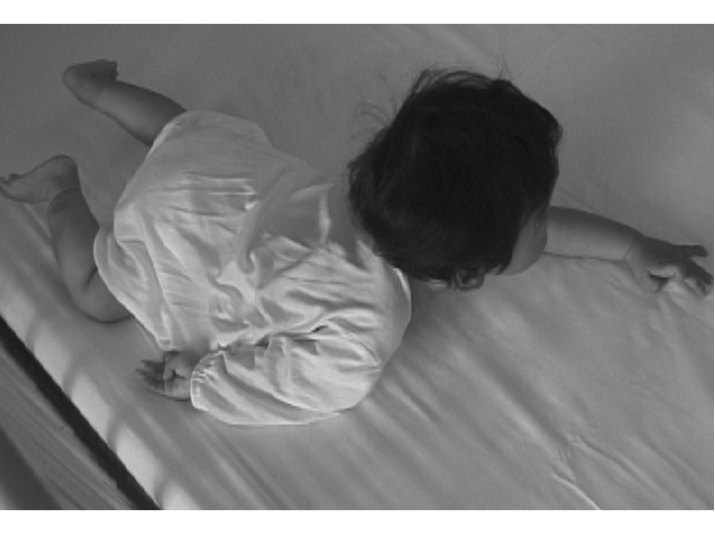
An eight-month-old boy with Erb's palsy at birth had no shoulder abduction or elbow flexion against gravity.

An 8 month old male infant with obstetric brachial plexus palsy associated with a breech delivery (at 40 weeks 1 day, birth weight: 3535 g), was treated by peripheral nerve transfer. He was complicated with phrenic nerve palsy, and a surgical treatment (reefing of the diaphragm) for this lesion had been undertaken at two months of age in another institute. At the first examination in our institute (at 5 months of age), active shoulder abduction and elbow flexion were absent (Fig. 1). Mental and other motor functional developments were normal. During 3 months of observation, no spontaneous recovery of elbow flexion or shoulder abduction was shown. On electrophysiological evaluations, no action potential of the neuromuscular unit was revealed in the biceps and deltoid muscles. The action potential of the neuromuscular unit of the abductor pollicis brevis muscle showed a normal wave. Physical and electrical examinations revealed an upper trunk type (C5-C6) right-side plexopathy. We considered the possibility of spontaneous recovery for several months, but functional recovery was poor. An Oberlin's nerve transfer and an accessory to suprascapular nerve transfer were selected to facilitate a rapid motor functional recovery of the biceps and deltoid muscles.

**Figure 2:**
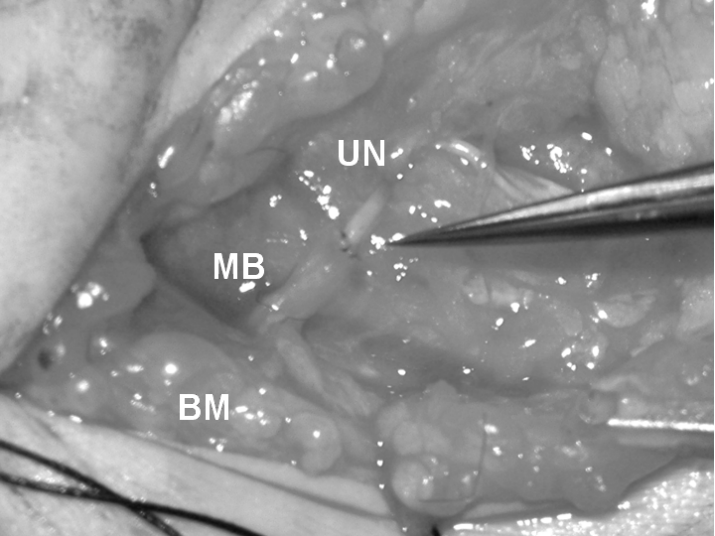
Intraoperative view of the partial ulnar nerve transfer to the musculocutaneous branch of the biceps muscle. The forceps indicate one fascicle of the ulnar nerve. UN; ulnar nerve. MB; motor branch of the musculocutaneous nerve. BM; biceps muscle.

Under general anesthesia, an operation was performed in the supine position. The brachial artery and the median, ulnar, and a branch of the musculocutaneous nerve supplying the biceps muscle were identified at a level approximately 7.0 cm distal from the acromion. One fascicle of the ulnar nerve was separated at the same level as the branch of the biceps muscle. We confirmed a fascicle corresponding to the motor fascicle of the ulnar nerve by microelectronic stimulation, and we then transferred this fascicle to the motor branch of the biceps muscle [1,2]. End-to-end nerve repair, at a level approximately 1.0 cm proximal from the insertion to the biceps muscle, was then performed using 10-0 sutures (Fig. 2). After resection of the omohyoid muscle, the upper trunks appeared. The spinal accessory nerve and the suprascapular nerve were identified, and the spinal accessory nerve was then transferred to the suprascapular nerve. The duration of these procedures was two hours and forty minutes. After surgery, the upper arm of the operative site was set free with no cast immobilization. No specific motor re-education program was used post-operatively.

A primary contraction of the biceps muscle appeared two months after nerve transfer (British Medical Research Council grading (MRC): M1). Full ranged elbow flexion (MRC: M4), and 90 degree shoulder abduction (MRC: M3), were obtained five months after surgery. Forty months after surgery, the ranges of both elbow flexion, M5, and shoulder abduction, M4, were full.. However, these muscles were somewhat weak compared with the contralateral site. Sensation in the ulnar nerve distribution in the hand was obscure, though the patient did not complain of any discomfort in the hand.

## Discussion

In 2002, the first two cases using Oberlin's nerve transfer (at 16 and 18 months after birth) for Erb's birth palsy of the C5-C6 type were reported by Al Quattan [3]. In 2004, Noaman et al. [4] followed this treatment method for 7 obstetric brachial plexus palsies with no elbow flexion (at 11 to 24 months after birth). This report supplements those reports and details a case younger (8 months after birth) than previous cases. Spontaneous recovery of obstetric brachial plexus palsy occasionally occurs; reported rates vary widely, ranging from 7 per cent to 96 per cent. Complete recovery can be expected only if the muscles start contracting by the first month [5]. Tassin [6] treated forty-four brachial plexus palsy cases due to obstetric lesion without surgical treatment; suggesting that primary surgical repair of the brachial plexus was warranted if recovery of the biceps had not began at three to four months of age, because in such cases functional prognosis was considered poor. Based on these reports, we performed peripheral nerve transfers at 8 months of age.

In our case, primary contraction of the biceps muscle appeared at 8 weeks after nerve transfer. In previous cases, initial biceps motor return was noted at 12 and 14 weeks, respectively, after surgery [1]. Motor reinnervation of the biceps muscle occurs within two to three months after partial ulnar nerve transfer, and thus elbow flexion is restored before permanent atrophy of the muscle occurs. In adult, Leechavengvongs et al. [2] performed partial ulnar nerve transfer for thirty-two patients with brachial plexus palsy. They reported that initial recovery was noted at 2 to 5 months after surgery (mean 3), and also described a functional recovery rate of 93% (patients achieving M4 or better), which compares favorably with other reported methods of brachial plexus neurotization [7]. Moreover, partial ulnar nerve transfer was possible to undertake with just one incision at the middle upper arm; whereas other procedures require many large incisions. A lower level of invasiveness is one of the advantages of this method, as well as motor reinnervation of the muscle being faster than other procedures.

Noaman et al. [4] noted four indications of partial ulnar nerve transfer for upper obstetric brachial plexus palsy; 1) breech delivery with avulsion of C5 and C6 nerve roots. 2) late presentation with good recovery of shoulder function. 3) spontaneous recovery of the upper obstetric brachial plexus palsy without biceps function, and 4) neuroma-in-continuity of the upper trunk, with good intraoperative shoulder muscle nerve condition, the same as a preoperative good shoulder function but with no biceps action. Our case would not have satisfied their criteria. The patient had not recovered elbow flexion and shoulder abduction at 8 months after birth; therefore, it was necessary to rapidly restore flexion of the elbow joint and to stabilize the shoulder joint. Stabilization of the shoulder joint was achieved by accessory to suprascapular nerve transfer. For restoration of the flexion of the elbow joint, there are several operations. Intracostal nerve transfer [7] is one of the procedures to restore flexion of the elbow joint. However, the patient in this case had had phrenic nerve palsy and an operation had already been performed. Intracostal nerve transfer for a patient with phrenic nerve palsy is a danger because a respiratory disturbance sometimes occurs post-operatively. Nerve grafting is a useful procedure for restoration of flexion of the elbow joint, but motor reinnervation comes later than in partial ulnar nerve transfer. Thus, we selected a partial ulnar nerve transfer despite the intercostal nerve transfer and nerve grafts.

## Conclusion

This case demonstrates an excellent result of an Oberlin's nerve transfer for restoration of flexion of the elbow joint in Erb's birth palsy. However, at this time partial ulnar nerve transfer for Erb's birth palsy is an optional procedure; a larger number of cases will need to be studied for it to be widely accepted as a standard procedure for Erb's palsy at birth.

**Figure 3:**
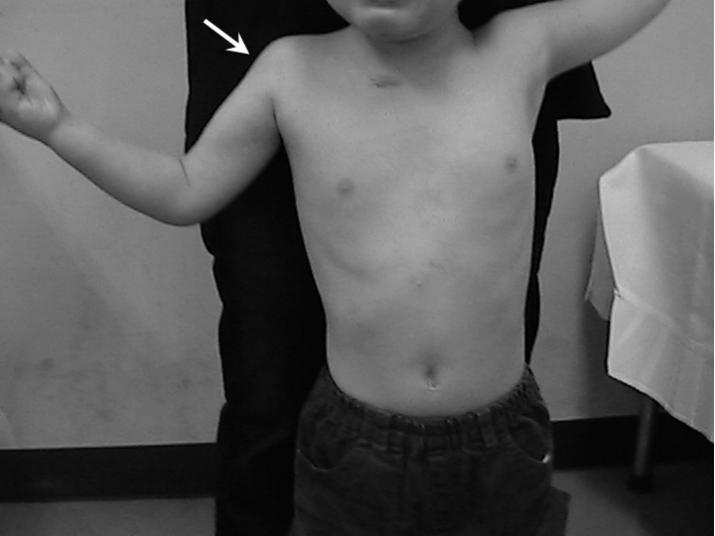
Forty months after surgery. The elbow joint can flex over a full range of active motion, the same as the contralateral side. The shoulder is stable, but atrophy of the deltoid muscle is seen (white arrow).
